# Vacuum-assisted closure device enhances recovery of critically ill patients following emergency surgical procedures

**DOI:** 10.1186/cc8193

**Published:** 2009-12-05

**Authors:** Stefano Batacchi, Stefania Matano, Alessandra Nella, Giovanni Zagli, Manuela Bonizzoli, Andrea Pasquini, Valentina Anichini, Valentina Tucci, Giuseppe Manca, Kevin Ban, Andrea Valeri, Adriano Peris

**Affiliations:** 1 Anaesthesia and Intensive Care Unit of Emergency Department, Careggi Teaching Hospital, Viale Morgagni 85, 50139, Florence, Italy; 2 Department of General Surgery, Careggi Teaching Hospital, Careggi Teaching Hospital, Viale Morgagni 85, 50139, Florence, Italy; 3 Department of Emergency Medicine, Beth Israel Deaconess Medical Center, Harvard Medical School, One Deaconess Road WCC-2, Boston, MA 02215 USA

## Abstract

**Introduction:**

Critically ill surgical patients frequently develop intra-abdominal hypertension (IAH) leading to abdominal compartment syndrome (ACS) with subsequent high mortality. We compared two temporary abdominal closure systems (Bogota bag and vacuum-assisted closure (VAC) device) in intra-abdominal pressure (IAP) control.

**Methods:**

This prospective study with a historical control included 66 patients admitted to a medical and surgical intensive care unit (ICU) of a tertiary care referral center (Careggi Hospital, Florence, Italy) from January 2006 to April 2009. The control group included patients consecutively treated with the Bogota bag (Jan 2006-Oct 2007), whereas the prospective group was comprised of patients treated with a VAC. All patients underwent abdominal decompressive surgery. Groups were compared based upon their IAP, SOFA score, serial arterial lactates, the duration of having their abdomen open, the need for mechanical ventilation (MV) along with length of ICU and hospital stay and mortality. Data were collected from the time of abdominal decompression until the end of pressure monitoring.

**Results:**

The Bogota and VAC groups were similar with regards to demography, admission diagnosis, severity of illness, and IAH grading. The VAC system was more effective in controlling IAP (*P* < 0.01) and normalizing serum lactates (*P* < 0.001) as compared to the Bogota bag during the first 24 hours after surgical decompression. There was no significant difference between the SOFA scores. When compared to the Bogota, the VAC group had a faster abdominal closure time (4.4 vs 6.6 days, *P* = 0.025), shorter duration of MV (7.1 vs 9.9 days, *P* = 0.039), decreased ICU length of stay (LOS) (13.3 vs 19.2 days, *P* = 0.024) and hospital LOS (28.5 vs 34.9 days; *P* = 0.019). Mortality rate did not differ significantly between the two groups.

**Conclusions:**

Patients with abdominal compartment syndrome who were treated with VAC decompression had a faster abdominal closure rate and earlier discharge from the ICU as compared to similar patients treated with the Bogota bag.

## Introduction

Intra-abdominal hypertension (IAH) is defined as a sustained pathological elevation in intra-abdominal pressure (IAP) above 12 mmHg [1,2]. The effect of persistent elevation of IAP beyond 20 mmHg is commonly referred to as abdominal compartment syndrome (ACS) resulting in depressed renal function, cardiac output, respiratory mechanics, and mesenteric perfusion [1-9]. Capillary leakage following the evolution of systemic inflammatory response syndrome in septic and trauma patients contributes to diminished abdominal wall compliance, as well as the need for mechanical ventilation and high positive end-expiratory pressures. The altered compliance of the abdominal wall is made worse by the increase in the intraabdominal volume (ileum, gastroparesis, capillary leakage, interstitial fluid loading), which frequently occurs in intensive care unit (ICU) patients as a consequence of major trauma, opioid infusions and/or parenteral nutrition. The incidence of IAH has been reported to be as high as 40% in post-surgical [6] and severely injured patients [10], and 30% in a population of medical and surgical ICU patients [11]. In particular, major trauma patients are at risk for increasing IAP and subsequently developing ACS.

The importance of IAP monitoring to prevent ACS in critically ill patients has been widely emphasized in the literature [1,3,6,12-17], even if routine IAP monitoring has yet to be made standard in many ICUs internationally [18].

The management of IAH includes both medical and surgical interventions [12]. The medical approach consists primarily of reducing intra-abdominal volume (nasogastric/colonic decompression, prokinetic drugs) or increasing compliance of the abdominal wall through neuromuscular blockade. Although non-surgical treatments must be attempted as the first step in the treatment of IAH, worsening IAP and/or deteriorating organ dysfunction requires surgical decompression with a temporary abdominal closure (TAC) system [12].

The aim of the present investigation was to evaluate the efficacy of two different TAC systems (Bogota bag and vacuum-assisted closure (VAC) device) in ICU patients requiring emergency open abdomen treatment.

## Materials and methods

### Study design and definitions

Our study group was recruited from the ICU of the emergency department of a tertiary care referral center (Careggi Teaching Hospital, Florence, Italy) from January 2006 to April 2009. During that time period, a total of 1350 patients were admitted to the ICU (trauma patients: 37.4%; medical: 35.3%; surgical: 27.3%). Among these, 66 patients (divided into a historical control and prospective group) were included in the study. Patient diagnoses included major trauma, sepsis, visceral and vascular surgery (gut perforation, gangrenous cholecystitis, aortic aneurysm rupture, and gut ischemia).

With regards to the prevention of ACS, our approach was to monitor the IAP of all patients admitted with abdominal or pelvic trauma, head trauma with intracranial hypertension, respiratory failure with high pressure support needs, major trauma patients requiring large volume fluid resuscitation, complicated abdominal surgery patients, and in all patients with a high likelihood of developing ACS based upon their clinical condition and laboratory values.

We monitored IAP using a urinary bladder pressure gauge (AbViser, Wolfe Tory Medical Inc., Salt Lake City, UT, USA) in supine patients until discharge or death [1]. Every four hours, 50 ml of saline solution was instilled in the urinary bladder closed system and the pressure was measured. In the VAC group, the IAP was not measured during system aspiration, which was set to intermittent. IAH was defined as a sustained pathological elevation in IAP of 12 mmHg or more, and graded in four classes as recommended [1]. IAH grading (Grade I: IAP 12 to 15 mmHg; Grade II: IAP 16 to 20 mmHg; Grade III: IAP 21 to 25 mmHg; Grade IV: IAP >25 mmHg) was compared with values measured after surgical decompression.

The open abdomen protocol was started at the end of the surgical phase: muscular fascia was sutured and the urinary bladder pressure was measured, under complete neuromuscular blockade. The limit of 12 mmHg was chosen as cut-off value for open abdomens [19]. In cases where the IAP was greater than 12 mmHg, stitches were removed starting from the middle of laparotomy incision, and the TAC was applied. Abdominal wall closure was performed when the IAP remained below 12 mmHg for 48 consecutive hours without neuromuscular blockade. All patients received full intensive care management, including antimicrobial therapy, mechanical ventilation, fluid resuscitation/transfusion and amines as necessary.

This study was designed as an observational, prospective study with a historical control group. Patients were divided in two groups: a historical control group (Bogota group), in which decompressive laparotomy was performed with placement of a Bogota bag (January 2006 to October 2007), and a prospective group (VAC group) after the adoption of VAC (Kinetic Concepts, Inc., San Antonio, TX, USA) in the organizational protocol (November 2007 to April 2009). The study was conducted in accordance with the principles of the Declaration of Helsinki, and was approved by our institutional ethics committee, which waived the need for informed consent in consideration of the nature of the study.

### Data management

Patient data were recorded in our ICU-database (FileMaker Pro 5.5v2, FileMaker, Inc, Santa Clara, CA, USA). For each patient, demographic, clinical characteristics and laboratory parameters were collected. Length of stay (LOS) in the ICU, Acute Physiology and Chronic Health Evaluation (APACHE) II, and Simplified Acute Physiology Score (SAPS) II were recorded.

To evaluate the efficacy of surgical decompression, the following data were compared: 1) IAP, indirectly evaluated by measuring urinary bladder pressure; 2) Sequential Organ Failure Assessment Score (SOFA); 3) arterial plasma lactates (mmol/L). IAP, SOFA score and lactates were collected sequentially: T0: before decompression; T1 to T24: every four hours during the first day after surgery; T24C: 24 hours after abdominal closure; TMS: at the end of monitoring.

All analyses were performed using MedCalc 10.1 statistical software package (MedCalc Software, Broekstraat 52, 9030 Mariakerke, Belgium). Continuous variables were analysed with analysis of variance or a two-tail Mann-Whitney test, and the results were expressed as the mean ± standard deviation (SD). Categorical variables were examined using Fisher's exact test. Multiple logistic regression has been performed on demographic and clinical parameters.

### Temporary abdominal closure systems

The VAC system consists of a polyurethane sponge cut to the appropriate size of the open-abdominal wound and placed over a sterile dressing. The sponge, with an 18-F suction tube, is placed on abdominal cavity and covered with second sterile adherent occlusive dressing. Suction is applied to the sponge using a portable pump. Advantages include maintenance of abdominal domain and elimination of temporary fascial suturing [20]. This dressing needs to be changed every 24 to 72 hours.

The Bogota bag utilizes a large sterile plastic bag, which is split open, to cover the abdominal viscera. The bag can be secured to either the skin or fascia. This system minimizes fluid loss, is easy to remove, relatively inexpensive, biologically inert and minimizes the future development of adhesions [13].

## Results

Demographic characteristics did not differ significantly between groups, as well as severity of illness scoring (SAPS II, APACHE II; Table 1). Male gender was predominant (77.3%), as was the total ICU admission of the study period (73.5%). Primary hospital admission diagnoses were abdominal/vascular pathology (36.4%), major trauma (33.3%), or abdominal sepsis (30.3%), equally distributed between the two groups. Grading of the IAH in each group was similar (Table 1). No device-related complications or secondary abdominal infections were observed. In all patients, protective ventilation was set to guarantee a tidal volume of 6 to 8 ml/Kg of predicted body weight, with a fraction of inspired oxygen of between 40% and 70% and a plateau pressure below 28 cm/H_2_O. Percutaneous dilatational tracheostomy was performed in 12 patients (38.7%) of the Bogota group and 15 patients (42.9%) of the VAC group. Five patients (16.1%) of the Bogota group and six patients (17.1) of the VAC group required continuous veno-venous hemodiafiltration. Two patients of the Bogota group (6.5%) and three (8.6%) patients of the VAC group developed a ventilator-associated pneumonia, which resulted in one fatality in the Bogota group.

**Table 1:** Baseline and clinical characteristics of patients treated with Bogota bag and with VAC device

		Overall (n = 66)	Bogota group (n = 31)	VAC group (n = 35)	*P*
**Age (years)**	*mean (SD)* *median (IQR)*	68.3 (23.8) 73 (57-79)	63.5 (21.8) 70 (42-79)	66.8 (18.2) 72 (63-79)	0.193
**Male sex, % (n)**		75.6% (50)	74.2% (23)	77.1% (27)	0.941
**BMI**	*mean (SD)* *median (IQR)*	25.6 (4.1) 24.7 (22.8-26.2)	25.9 (7.1) 25 (23-27)	26.1 (7.5) 25 (22-26)	0.271
**SAPS II score**	*mean (SD)* *median (IQR)*	51.8 (12.8) 51 (38-67.5)	49.1 (17.5) 40 (32-65)	52.4 (17.8) 51 (43-68)	0.274
**APACHE II score**	*mean (SD)* *median (IQR)*	22.7 (2.3) 21 (17-27)	23.2 (7.1) 23 (18-30)	21.6 (6.5) 21 (16-26)	0.298
**Hospital admission diagnosis, % (n):**					
*Abdominal/vascular pathology*		36.4% (24)	35.5% (11)	37.1% (13)	0.951
*Major trauma*		33.3% (22)	38.7% (12)	28.6% (10)	0.439
*Abdominal sepsis*		30.3% (20)	25.8% (8)	34.3% (12)	0.592
**Grades of IAH, % (n):**					
*Grade 1 (12-15 mmHg)*		40.9% (27)	45.1% (14)	37.1% (13)	0.617
*Grade 2 (16-20 mmHg)*		31.8% (21)	29.1% (9)	34.3% (12)	0.792
*Grade 3 (21-25 mmHg)*		21.2% (14)	19.4% (6)	22.9% (8)	0.772
*Grade 4 (>25 mmHg)*		6.1% (4)	6.4% (2)	5.7% (2)	0.931
**Length of open** abdomen (days)*	*mean (SD)* *median (IQR)*	5.8 (3.3) 5 (3-7)	6.6 (3.7) 6 (3-8)	4.4 (1.8) 4 (3-5)	0.025
**Duration of MV** (days) *	*mean (SD)* *median (IQR)*	8.4 (6.1) 5 (3-13)	9.9 (6.5) 8 (3-14)	7.1 (5.4) 5 (3-12)	0.039
ICU LOS (days) *	*mean (SD)* *median (IQR)*	14.1 (8.2) 9 (5-22)	19.2 (9.6) 16 (7-36)	13.3 (5.2) 6 (4-17)	0.024
**Total hospital** LOS (days)*	*mean (SD)* *median (IQR)*	31.9 (6.5) 24 (16-38)	34.9 (8.8) 29 (22-45)	28.5 (4.7) 21 (14-35)	0.019
**Intra-ICU mortality, % (N)**		24.2% (16)	29% (9)	20% (7)	0.565
**Intra-hospital mortality, % (N)**		28.8% (19)	35.4% (11)	22.9% (8)	0.288

Calculations of ICU LOS and length of open abdomen were performed excluding deceased patients from each group. Grading of IAH of each group was assessed after surgical decompression.

Data are expressed as mean (SD) and median (IQR 25^th ^to 75th). Percent data are referred to the total population of each group. Statistical analysis: two-tailed Mann-Whitney test and two-tailed Fisher's exact test. *P < 0.05.

APACHE = Acute Physiology And Chronic Health Evaluation; BMI = body mass index; IAH = intraabdominal hypertension; ICU = intensive care unit; IQR = interquartile range; LOS = length of stay; MV = mechanical ventilation; SAPS = Simplified Acute Physiology Score; SD = standard deviation; VAC = vacuum-assisted closure.

The mean fluid aspiration from the VAC system during the first 24 hours was 820 ± 255 ml. Prior to the VAC removal, mean drainage was 180 ± 80 ml. Conversely, in the Bogota group, passive drainage was achieved through surgical tubes. Mean fluid collection in the first 24 hours was 430 ± 190 ml and less than 100 ml before abdominal closure. The difference in the first 24 hours of drainage was found to be significant (*P *= 0.0243). No significant technique-specific complications in TAC systems used occurred. In three patients, the VAC substitution was performed early due to external air aspiration due to an improperly placed occlusive dressing. Eight patients in the Bogota group (25.8%) and six patients in the VAC group (17.1%) underwent surgical re-exploration due to suspicion of gut ischemia. In all these cases, the TAC devices were re-applied.

Primary closure without placement of the TAC system change was possible in four patients (13%) in the Bogota group and nine patients (26%) in the VAC group. Overall, the VAC system permitted earlier abdominal closure as compared with the Bogota bag. Indeed, patients in the VAC group required fewer days of open abdomen treatment (4.4 vs 6.6 (mean), 4 vs 6 (median); *P *= 0.025) compared with the Bogota group. Weaning patients from mechanical ventilation occurred more quickly in patients from the VAC group (7.1 vs 9.9 (mean); 5 vs 8 (median); *P *= 0.039; Table 1).

The ICU and total hospital LOS reflected the differences in open abdomen time and duration in mechanical ventilation observed among both groups. Patients treated with the VAC system showed significantly shorter mean and median ICU (13.3 and 6 days, respectively; *P *= 0.024) and hospital LOS (28.5 and 21 days, respectively; *P *= 0.019) than patients treated with the Bogota bag (Table 1). Intra-ICU mortality rates were not significantly different between the two groups. Similarly, total hospital mortality was lower for the VAC patients (31.4% vs 41.9%), but was not statistically significant (Table 1).

**Figure 1:**
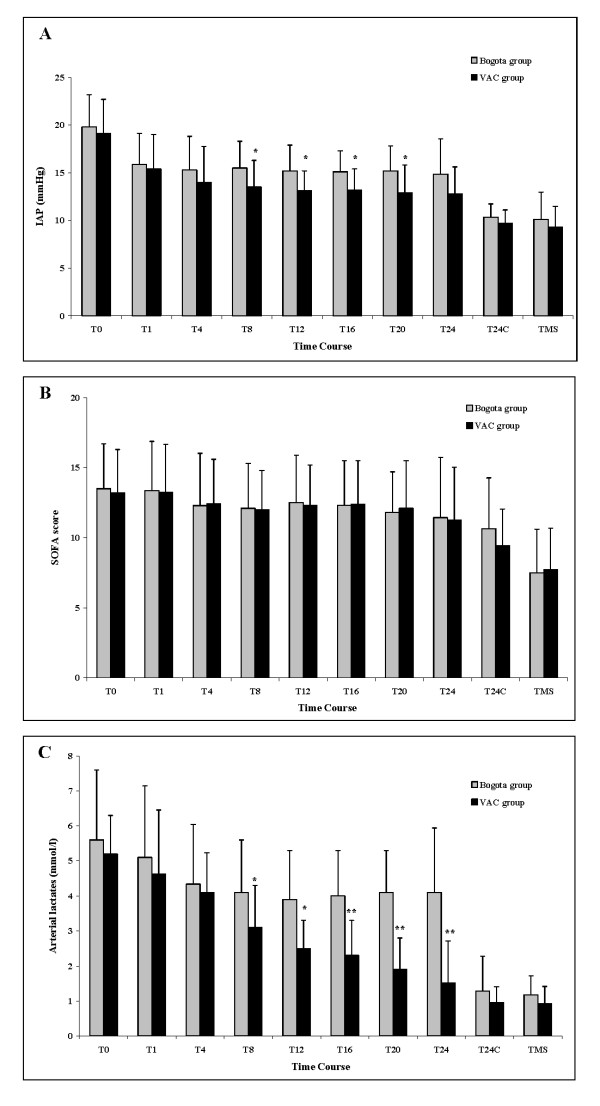
Comparison of **(a)** IAP, **(b)** SOFA score, and **(c)** arterial lactates between patients treated with Bogota bag (Bogota group) and patients treated with VAC device (VAC group). Data are represented as mean ± standard deviation. Statistical analysis was a two-tailed Mann-Whitney test. *P < 0.01; **P < 0.001. IAP = intraabdominal pressure; SOFA = Sequential Organ Failure Assessment; T0 = before surgical treatment; T1-24 = first day (collected every four hours) after surgical treatment; T24C = 24 hours after abdomen closure; TMS = at monitoring suspension; VAC = vacuum-assisted closure.

**Figure 2:**
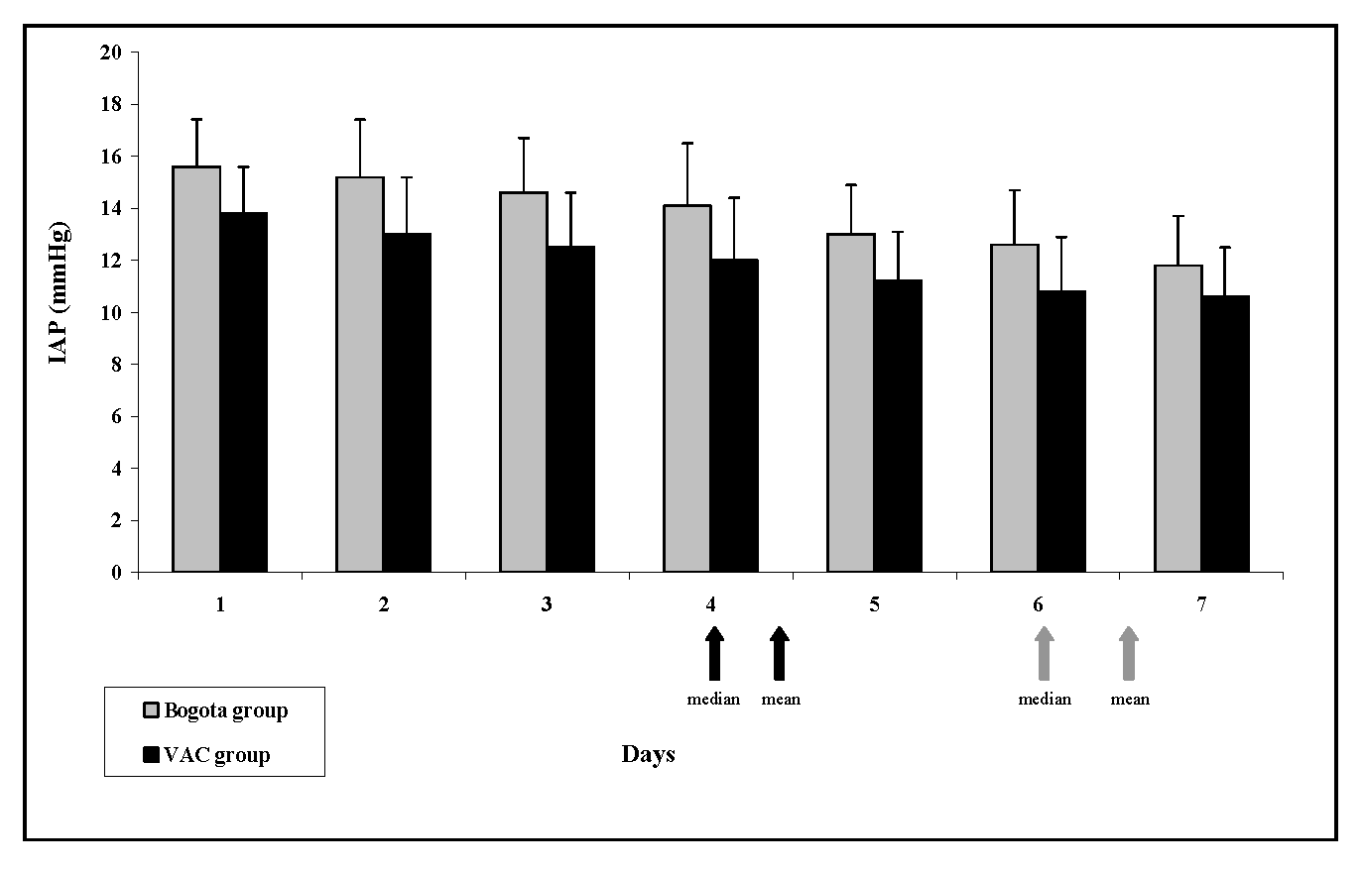
Daily IAP evolution during the first seven days after decompression in Bogota group and VAC group. Data represent mean ± standard deviation. Grey arrows: median/mean time of abdominal closure in Bogota group. Black arrows: median/mean time of abdominal closure in vacuum-assisted closure (VAC) group. IAP = intraabdominal pressure.

Figure 1 illustrates the comparison of the IAP (Figure 1a), SOFA score (Figure 1b) and serial arterial lactates (Figure 1b) between the Bogota and VAC groups. The time course of the IAP measurements showed a significant faster decrease between the 8th and 24th hour after decompression in the VAC group with respect to the Bogota group (*P *< 0.01). After abdominal closure, this difference was not significant (Figure 1a). Conversely, the SOFA score improved in a time-dependent manner in both groups, without significant differences (Figure 1b). Finally, arterial lactates mirrored the trend in reduction observed for IAP, decreasing faster in the VAC group than in the Bogota group between the 8th and 24th hour after decompression (*P *< 0.001; Figure 1c). Figure 2 shows the daily IAP measurements in the two groups during the first week after surgical decompression.

With regards to the differences between survivors and non-survivors, contingency analysis showed that the significant relative risk (RR) for death were identified in age older than 70 years (RR 2.9), IAP value above 20 before decompression (RR 3.4), pre-operative lactates level above 8 (RR 2.8) and post-operative lactate levels above 6 (RR 3.2). Multiple regression analysis confirmed that variables predicting the intra-ICU mortality were age (*P *= 0.047), IAP values after surgical decompression (*P *= 0.026) and both pre- and post-surgical lactates level (*P *= 0.032 and *P *= 0.022, respectively). Conversely, SAPS II and APACHE II scores did not provide a statistically significant mortality prediction in our patient population.

## Discussion

This study addresses the role of two surgical interventions in controlling IAP. The need and effectiveness of non-invasive strategies to control the IAP, such as opioid limitation, prokinetic and gut decompression, should be evaluated daily [12]. However, when IAP increases, the overuse of non-invasive treatments may lead to complications (e.g. prolonged neuromuscular blockade can produce a delay in mechanical ventilation weaning and is associated with an increased risk of critical illness polyneuropathy/myopathy development) making the overall care of the patient more problematic.

Several TAC systems are available for open abdomen management. Some use negative pressure techniques (VAC and vacuum pack) while others do not require active aspiration (Bogota bag, Wittmann patch, Dynamic retention sutures, absorbable or non-absorbable mesh or sheet). A recent systematic review [21] underscores the need for randomized trials to produce comparative data. The literature suggests that the VAC device and the Wittmann patch were the TAC systems with the lowest associated mortality rates and higher closure success rates [21]. A recent survey of the American Association for the Surgery of Trauma found that in almost 60% of patients with increased abdominal pressures requiring surgical intervention, temporary closure was made possible using vacuum devices, compared with only 18% who used the Bogota bag [22]. Unfortunately, the response rate in that study was only 26% and no definitive conclusions on optimal management strategies for the open abdomen can be made.

This is the first study in which the Bogota bag and the VAC system have been compared in the ICU setting [9]. The main finding observed in our sample was the superiority of the VAC device in controlling IAP, permitting a shorter duration of open-abdomen maintenance with risk-related reduction, mechanical ventilation and, consequently, ICU/hospital LOS (Table 1). Increased success is likely to be attributable to the active and adjustable negative pressure afforded by the pump of the VAC device, with aspiration of post-surgical/inflammatory/fluid load edema and of peritoneal exudates. Although the Bogota bag is less expensive and provides a sterile cover for mechanical protection of the intra-abdominal organs, it does not permit active drainage. In our study, fluid drainage during the first 24 hours after decompression was significantly higher with the VAC system than drainage associated with the Bogota bag (820 ml vs 430 ml; *P *= 0.0243). This may explain the faster decrease in IAP observed in the VAC group (Figures 1a and 2).

Normal IAP has been defined as 5 to 7 mmHg in critically ill adult patients [1]. The choice to use the TAC device with an IAP of 12 mmHg was based on work performed by Malbrain and colleagues in 2006 [1]. This choice may represent a limitation of our study because a low cut-off value may have enrolled patients in our study who would not have progressed to ACS. However, even lower levels of IAP have been associated with the development of intra-abdominal complications. Gargiulo and colleagues showed that gut bacterial translocation increased with an IAP of only 10 mmHg in experimental models [23]. The possible mechanism involved in bacterial translocation is thought to be the loss of the mucosal barrier due to the reduction in mesenteric blood flow [24]. A IAP value of 12 mmHg appears to be appropriate in avoiding reductions in renal blood flow and function reported with IAP values of 15 mmHg in both human and experimental models [25,26].

The overall hospital mortality rate in our study was lower than reported in a recent review (30% vs 50%) [27]. This notable difference could be explained by the 12 mmHg cut-off value chosen for the use of a temporary abdominal closure device. Nevertheless, most of the studies reviewed by De Waele and colleagues [27] did not use an active clearance device after surgical decompression. We believe the lower mortality rate is due to the early use of the VAC device. The systematic review of Boele van Hensbroek and colleagues shows that mortality rates in eight studies on the VAC technique vary between 7% and 38%, with a mean of 18% [21]. Instead, the mortality rate when using the Bogota bag was between 18% and 53% in three retrospective studies. Based on the limited data found in the literature and despite the limitations described in our study, the use of the VAC device may be associated with a lower mortality rate.

This study's lack of randomization should be considered as another limiting factor because the study design did not permit us to exclude patients enrolled after the introduction of the VAC-system. These patients could have had a better response to their treatment regardless of the decompression system used. Finally, our sample size was not large enough to determine if the mortality rate can be influenced by the use of VAC therapy.

## Conclusions

Patients with abdominal compartment syndrome who were treated with VAC decompression had a faster abdominal closure rate and earlier discharge from the ICU as compared with similar patients treated with the Bogota bag.

## Key messages

• The use of the VAC device is associated with faster abdominal closure, lower duration of mechanical ventilation, and decreased ICU/hospital LOS.

• The VAC system seems to have a role in lowering the mortality rate, but further studies are necessary.

## Abbreviations

ACS: abdominal compartment syndrome; APACHE: acute physiology and chronic health evaluation; IAH: intraabdominal hypertension; IAP: intraabdominal pressure; ICU: intensive care unit; LOS: length of stay; RR: relative risk; SAPS: simplified acute physiology score; SD: standard deviation; SOFA: Sequential Organ Failure Assessment Score; TAC: temporary abdominal closure; VAC: vacuum-assisted closure.

## Competing interests

The authors declare that they have no competing interests.

## Authors' contributions

AP, MB, AP designed the study. AP, MB, SM, AN and SB reviewed the literature. AN, SM, TV and VA collected and elaborated the data. GM and AV performed surgical interventions. AN, GZ and AP wrote the manuscript. KB revised statistics and language. All authors have revised the manuscript.
